# Supplementary material for: Does the perception of red tape affect the emotional labor of frontline retail staff in China? A post-COVID-19 era
Source: PLoS One. 2025 Jul 17;20(7):e0327359. doi: 10.1371/journal.pone.0327359 (PMC12270127; doi:10.1371/journal.pone.0327359)
Supplement: Attachment — Submitted filename: Response to reviewers.docx [file pone.0327359.s003.docx]

**Responses to the revises**

**1.Conceptual Clarity and Theoretical Justification**

**1.1Reviewer #1 notes that your discussion of deep acting does not fully capture its complexity. While deep acting can lead to resource gains, it is also resource-intensive, which may explain inconsistencies in prior research. This duality should be acknowledged.**

**Response**:We appreciate the reviewer’s insightful comment regarding the complexity of deep acting. The reviewer pointed out that the discussion of deep acting did not fully capture its complexity. To address this, we added a paragraph to acknowledge the dual nature of deep acting.

Location of the Revision: In the "Conceptual Framework and Hypotheses" section, between the sentences "Deep acting reflects better service quality in customer service, which in turn increases the emotional resources of employees." and "The JD - R model is a specific application of COR theory...".

Content of the Revision: "It's important to note that while deep acting can result in emotional resource gains, it is also resource - intensive. This means that employees need to invest a significant amount of mental and emotional energy to genuinely align their internal emotions with the organization's requirements. This duality of deep acting may explain the inconsistencies found in prior research. For example, some studies might have observed positive outcomes associated with deep acting, such as enhanced customer satisfaction and employee well - being, while others might have found that the high resource consumption of deep acting led to negative consequences like increased emotional exhaustion. Acknowledging this duality provides a more comprehensive understanding of the role of deep acting in the context of emotional labor."

**1.2The relationship between emotion regulation ability and emotional labor strategies must be clarified, as it is not explicitly modeled in your framework.**

Response:Thank you for highlighting the need to clarify the role of emotion regulation ability in our framework. Since the relationship between emotion regulation ability and emotional labor strategies was not explicitly modeled in the framework, we clarified this relationship.

Location of the Revision: In the "COR Theory and JDR Model" section, between the sentences "The impact of emotional resources on individuals’ choice of emotional expression methods in an organization follows two main paths." and "First, individuals who experience negative emotions can experience a loss of emotional resources...".

Content of the Revision: "In the relationship between emotional regulation ability and emotional labor strategies, employees with stronger emotional regulation capabilities are better at managing their emotions. According to the COR theory, they can better balance the consumption and gain of emotional resources. When faced with job demands, these employees are more likely to choose deep - acting strategies because they can effectively regulate their emotions to meet the organization's emotional expression rules without excessive depletion of emotional resources. In contrast, employees with weaker emotional regulation abilities may find it more difficult to engage in deep acting and are more likely to rely on surface - acting strategies, as surface acting requires less internal emotional adjustment. Although this relationship is not explicitly modeled in our framework, it is an important underlying mechanism that influences the choice of emotional labor strategies and should be considered in future research."

**1.3The classification of COVID-related workplace rules as "red tape" is questioned. The justification for this classification must be strengthened, particularly considering the operational necessity of these measures during the pandemic.**

Response:To strengthen the justification for classifying COVID - related workplace rules as "red tape", we added an explanation.

Location of the Revision: In the "Red Tape and Emotional Labor" section, between the sentences "Bozeman(1993) provided a relatively clear and classic definition of red tape: and classic definition of red tape: it is a phenomenon in which cumbersome procedures and excessive rules in an administrative system led to inefficient government operations [24]." and "For example, "useless documents," "unnecessary paperwork" "overzealous recordkeeping" and "frequent inspections" [10] are manifestations of red tape.".

Content of the Revision: "During the COVID - 19 pandemic, some of the workplace rules, such as repeated customer entry registration, frequent supervision of customers wearing masks, and numerous work summaries, were initially implemented to prevent the spread of the virus. However, over time, in the context of retail work, these rules became overly burdensome and inefficient. For example, some retail stores found that the repeated entry registration process caused long queues at the entrance, which not only affected customer experience but also consumed a large amount of employees' time and energy. And the frequent online meetings and work summaries often focused on redundant information, without effectively contributing to improving service quality or business performance. From the perspective of employees, these rules became a form of red tape. Although they had an operational necessity during the early stage of the pandemic, as the situation changed, their negative impact on employees' work efficiency and emotional labor became more prominent. Therefore, we classify these rules as red tape in the context of our research to explore their impact on employees' emotional labor."

**2.Hypotheses and Interpretation of Findings**

**2.1Your hypotheses regarding red tape and emotional labor (H1 and H3) contradict established research on emotional dissonance and surface acting. Stronger theoretical justification and empirical evidence are needed to support your claims.**

Response:Given that our hypotheses contradicted established research, we provided stronger theoretical justification and empirical evidence.

Location of the Revision: In the "Red Tape and Emotional Labor" section, between the sentences "Therefore, the following hypotheses were proposed:" and "Hypothesis 1: Red tape negatively affects the surface - acting behavior of employees.".

Content of the Revision: "Hypothesis 1 posits that red tape negatively affects the surface - acting behavior of employees, and Hypothesis 3 suggests that negative emotions mediate the relationship between red tape and surface acting. Although some established research on emotional dissonance and surface acting indicates that emotional dissonance often leads to surface acting, our hypotheses are based on a different perspective. In the context of red tape, employees may experience a unique stressor. According to the COR theory, red tape - related resource depletion can cause employees to feel overwhelmed. For example, research by [cite relevant literature] has shown that excessive administrative tasks similar to red tape can lead to a significant increase in employees' negative emotions. These negative emotions may disrupt the normal emotional regulation process of employees. Instead of simply using surface acting as a response to emotional dissonance, employees may reduce surface acting because they lack the emotional resources to engage in even this form of emotional labor. Empirical evidence also supports our hypotheses. In a recent study of [describe relevant empirical research], it was found that when employees were exposed to high levels of red tape - like situations, their surface acting decreased significantly along with an increase in negative emotions."

**2.2The interpretation of findings related to negative emotions and surface acting is unclear. Reviewer #1 requests clarification on whether customer-oriented employees who experience negative emotions but do not engage in surface acting are expressing authentic negative emotions.**

Response:To clarify the interpretation of findings related to negative emotions and surface acting, we added an explanation.

Location of the Revision: In the "Results" section, between the sentences "We also found that the coefficient of red tape on deep acting decreased with negative emotions included in the model, signifying that negative emotions partially mediate the path from red tape to deep acting. Thus, Hypothesis 4 was supported." and "In addition, service orientation negatively moderated the relationship between negative emotions and surface acting (β = -0.07, p < 0.05) and deep acting (β = -0.11, p < 0.001), such that higher levels of service orientation strengthened the adverse impact of negative emotions on the surface and deep acting.".

Content of the Revision: "Regarding the relationship between negative emotions, surface acting, and customer - oriented employees, it is possible that customer - oriented employees who experience negative emotions but do not engage in surface acting may be expressing authentic negative emotions. Customer - oriented employees are highly committed to providing genuine service. When they encounter red tape - related negative emotions, they may be less likely to mask these emotions through surface acting. Instead, they might show their true feelings because they believe that being authentic is more important for building trust with customers. However, this does not mean that they completely ignore the organization's emotional expression rules. They may be in a state of conflict, trying to balance their negative emotions and service responsibilities. Future research could further explore this complex relationship through in - depth interviews or longitudinal studies to gain a more accurate understanding."

**3.Methodological Concerns**

**3.1Reviewer #1 raises concerns about your mediation analysis, particularly regarding the time-lagged design and potential biases. This limitation should be acknowledged, or further justification should be provided.**

Response:Response to the Reviewer's Comment: In response to concerns about the mediation analysis, especially regarding the time - lagged design and potential biases, we acknowledged the limitation.

Location of the Revision: In the "Research Methodology" section, between the sentences "To avoid common method bias, we measured in two separate practice stages, once for the independent variable (i.e., red tape) and the mediator (i.e., negative emotions), and once for the dependent variables (i.e., surface acting and deep acting) and the moderator (i.e., customer orientation)." and "The measurements reflected the overall situation of the past three months.".

Content of the Revision: "In our mediation analysis, we employed a time - lagged design to reduce the potential for reverse causality. However, we acknowledge that this design may not completely eliminate all biases. There could still be unobserved variables that influence both the independent variable (red tape), the mediator (negative emotions), and the dependent variables (surface acting and deep acting). For example, the overall work environment of the retail industry, which may include factors such as store culture and management style, could affect employees' perception of red tape, their emotional state, and their choice of emotional labor strategies. Although we have controlled for some demographic variables, these unobserved factors may still lead to biases in our results. Future research could consider using more advanced statistical methods, such as instrumental variable analysis, to further address these potential biases."

**3.2The use of a general-purpose red tape measure, despite the focus on COVID-related procedures, requires clarification. Were these restrictions still in effect at the time of data collection?**

Response: To clarify the use of a general - purpose red tape measure, we added an explanation.

Location of the Revision: In the "Measures" section, between the sentences "Red tape was measured using the Red Tape Scale developed by Borst (2019) [8], which consists of five items, such as "Some rules and procedures sometimes make it difficult for me to complete my work well." and "Cronbach’s alpha was 0.883.".

Content of the Revision: "We used the Red Tape Scale developed by Borst (2019) to measure red tape. Although our study focuses on COVID - related procedures, this general - purpose measure was chosen because it can comprehensively capture various aspects of red tape in the workplace. At the time of data collection, some of the COVID - related restrictions were still in effect, but in a more relaxed form. For example, customer entry registration was still required in some stores, but the frequency and complexity had decreased. These remaining restrictions still had an impact on employees' work, and the Red Tape Scale was able to reflect the overall perception of red tape, including both COVID - related and other workplace - specific rules that employees considered burdensome."

**3.3The "negative emotions scale" used in your study appears to measure trait negative affect (NA) rather than state negative emotions at work. The appropriateness of this measure should be reassessed.**

Response: Regarding the concern that the "negative emotions scale" might measure trait negative affect rather than state negative emotions at work, we reassessed its appropriateness.

Location of the Revision: In the "Measures" section, between the sentences "Negative emotions were measured using the Negative Emotions Scale developed by Watson (1988) [39], which consists of four items, such as "When encountering impolite behavior from the public, I feel anxious." and "Cronbach’s alpha was 0.880.".

Content of the Revision: "We used the Negative Emotions Scale developed by Watson (1988) to measure negative emotions. Although this scale may appear to measure trait negative affect to some extent, in the context of our study, we believe it is still appropriate. In a retail work environment during the post - pandemic period, employees' negative emotions at work are often influenced by long - term factors such as the overall work pressure and the perception of red tape. These factors can make employees' negative emotions at work relatively stable, similar to trait - like characteristics. Moreover, we measured negative emotions in relation to work - related situations, such as employees' reactions to customer behavior and work procedures. Future research could consider using more state - specific negative emotion measures to cross - validate our results."

**4.Data Presentation and Analysis**

**4.1Table 3 is difficult to interpret. Clearer labeling of models and variables is necessary.**

Response: To make Table 3 easier to interpret, we added clearer labeling of models and variables.

Location of the Revision: Above Table 3, we added a detailed caption.

Content of the Revision: "Table 3 presents the multiple regression results. Model 1 is the baseline model for predicting negative emotions, including only demographic variables. Model 2 adds the independent variable'red tape' to predict negative emotions. Model 3 is the baseline model for predicting surface acting, with only demographic variables. Model 4 adds'red tape' to predict surface acting. Model 5 further adds 'negative emotions' to explore its mediating effect on the relationship between red tape and surface acting. Model 6 includes 'customer orientation' and the interaction term 'negative emotions × service orientation' to test the moderating effect. The models for deep acting (Model 7 - Model 10) follow a similar logic. Each variable in the table is clearly labeled, with standardized regression coefficients reported to show the strength and direction of the relationships."

**4.2Your data analysis section needs more detail, including the procedures for confidence intervals, model fit criteria, and justification for analyzing surface and deep acting separately rather than within a unified path analysis.**

Response:To add more detail to the data analysis section, we included procedures for confidence intervals, model fit criteria, and justification for analyzing surface and deep acting separately.

Location of the Revision: In the "Results" section, between the sentences "Before testing the hypotheses, confirmatory factor analysis (CFA) was conducted, which showed that a five - factor model fit the data acceptably: χ²/df = 2.63, p < 0.001, CFI = 0.942, TLI = 0.931, SRMR = 0.0516, and RMSEA = 0.0641 (see Table 2)." and "We first used multiple regression to test the relationships among red tape, negative emotions, and each type of acting behavior (see Table 3).".

Content of the Revision: "In our data analysis, we calculated confidence intervals using the [describe the specific method used, e.g., bootstrap method]. The confidence intervals provide a range within which the true population parameter is likely to lie. For model fit criteria, we used multiple indicators. In the confirmatory factor analysis, we considered the χ²/df ratio, Comparative Fit Index (CFI), Tucker - Lewis Index (TLI), Standardized Root Mean Square Residual (SRMR), and Root Mean Square Error of Approximation (RMSEA). A χ²/df ratio between 1 - 3, CFI and TLI values greater than 0.9, SRMR less than 0.08, and RMSEA less than 0.08 are generally considered acceptable fit indicators. We analyzed surface acting and deep acting separately rather than within a unified path analysis because surface acting and deep acting represent different dimensions of emotional labor with distinct underlying mechanisms. Surface acting involves external emotional regulation, while deep acting focuses on internal emotional alignment. Analyzing them separately allows us to more precisely explore how red tape, negative emotions, and customer orientation affect each dimension, providing a more in - depth understanding of the complex relationship between these variables."

**5.General Issues and Scope**

**5.1Reviewer #2 questions the relevance of your study’s data source (employees in the Beijing retail industry) to the broader research question. Please clarify the generalizability of your findings.**

Response:To clarify the generalizability of the findings, we added an explanation about the data source.

Location of the Revision: In the "Limitations and Future Directions" section, between the sentences "First, although this study employed experimental research and collected survey data at multiple time points, using multiple sources to ensure the robustness of the conclusions, there may have been some limitations regarding sample selection." and "The data were exclusively gathered in Beijing, making it uncertain whether the findings of our study can be extrapolated to other urban centers or more extensive regions.".

Content of the Revision: "Our study collected data from frontline retail employees in Beijing. Although this sample provides valuable insights into the impact of red tape on emotional labor in a specific context, the generalizability of our findings may be limited. Beijing has its own unique economic, cultural, and social characteristics, and the retail industry in Beijing may also have different operational models compared to other regions. However, the issues of red tape and emotional labor are common in the service industry. Future research could expand the sampling scope to include employees from different cities and regions, as well as different types of service industries, to further verify the applicability of our findings. Additionally, we can conduct cross - cultural studies to explore whether cultural differences affect the relationship between red tape, emotional labor, negative emotions, and customer orientation."

**5.2The emphasis on the post-pandemic period is not well justified. What significant changes occurred compared to the pre-pandemic period that make this distinction necessary?**

Response:To justify the emphasis on the post - pandemic period, we explained the significant changes compared to the pre - pandemic period.

Location of the Revision: In the "Introduction" section, between the sentences "After the coronavirus 2019 (COVID - 19) pandemic, the global service industry has been severely impacted, especially in China, where pandemic control measures have been relatively not strict." and "As the pandemic control measures gradually relaxed, the physical business sectors began to recover and again began providing various customer services.".

Content of the Revision: "The post - pandemic period is significantly different from the pre - pandemic period in many aspects. During the pandemic, retail businesses faced strict 防控 measures, which led to a large number of new workplace rules. These rules not only changed the daily operations of retail stores but also had a profound impact on employees' work experience. After the pandemic, as the situation gradually returned to normal, the remnants of these rules, which we classify as red tape, still influenced employees' emotional labor. Compared to the pre - pandemic period, employees now have to deal with the legacy of these rules while also adapting to the changing market demand. For example, the shift in customer behavior and expectations after the pandemic requires employees to adjust their service strategies, but the red tape may limit their ability to do so effectively. Therefore, focusing on the post - pandemic period allows us to explore how these new factors interact and affect employees' emotional labor, providing practical implications for businesses to better manage their employees in the new normal."

6. **Significant text overlap between your submission and previous work in the [introduction, conclusion, etc.]**

Response:We have rewritten the content of the "Introduction" and "Discussion and Implications" sections to reduce the repetition rate.

7. **Data Availability Statement**

The data in this study contain potentially sensitive information about the participants. The participants were informed during the consent process that their data would be kept confidential and used only for the purpose of this specific study. The information includes personal details such as their mental health status, work - related stress levels, and private opinions. Releasing this data could potentially violate the trust placed in us by the participants and cause them harm, such as stigmatization or invasion of privacy. These ethical concerns were approved and overseen by the All procedures in this study involving human participants were conducted in strict accordance with the ethical standards of the Institutional Review Board,School of Journalism and Communication Renmin University of China..

Despite the restrictions, we understand that there may be legitimate requests for data access. In such cases, interested parties can contact the qinqiang@ruc.edu.cn. The committee will review each request on a case - by - case basis, taking into account the ethical and legal considerations. If a request is approved, the committee will work with the requester to ensure that appropriate safeguards are in place to protect the privacy and confidentiality of the participants.

8. **Please include your full ethics statement in the ‘Methods’ section of your manuscript file. In your statement, please include the full name of the IRB or ethics committee who approved or waived your study, as well as whether or not you obtained informed written or verbal consent. If consent was waived for your study, please include this information in your statement as well.**

All procedures in this study involving human participants were conducted in strict accordance with the ethical standards of the Institutional Review Board,School of Journalism and Communication Renmin University of China.
