# Supplementary material for: Does the perception of red tape affect the emotional labor of frontline retail staff in China? A post-COVID-19 era
Source: PLoS One. 2025 Jul 17;20(7):e0327359. doi: 10.1371/journal.pone.0327359 (PMC12270127; doi:10.1371/journal.pone.0327359)
Supplement: Attachment — Submitted filename: rebuttal letter.docx [file pone.0327359.s004.docx]

rebuttal letter

Dear Editors and Reviewers,

We sincerely appreciate the thoughtful and constructive feedback provided on our manuscript entitled "Does the perception of red tape affect the emotional labor of frontline retail staff in China? A post-COVID-19 era". We have carefully revised the manuscript in accordance with the reviewers’ comments and believe the changes have significantly strengthened the theoretical clarity, methodological transparency, and empirical interpretation of our work.

Below, we offer a point-by-point response to each reviewer comment, indicating where and how revisions have been made.

**1.Clarify Theoretical and Conceptual Presentation. While the relationship between emotion regulation ability and emotional labor strategies is briefly discussed, it is suggested that this connection be visually incorporated into the theoretical framework to enhance the logic. Additionally, the applicability of 'red tape' in business contexts could be further elaborated. A comparison between different types of organizations (e.g., large chains vs. small retailers) may improve the explanatory power and generalizability of the study."**

Response:Thank you for this insightful suggestion. We have made several modifications to clarify our theoretical framework:

We have updated Figure 1 to incorporate “Emotion Regulation Ability” as a conceptual element. Though not statistically modeled, it is now visually presented as a dashed-line construct influencing the choice of emotional labor strategies, based on conservation of resources (COR) theory.

Textual Explanation of the Visual Addition

We revised the paragraph under “COR Theory and JD-R Model” to reference this addition:

“Although this relationship is not explicitly tested in our statistical models, we now incorporate it visually in the theoretical framework (see Figure 1) to illustrate how emotional regulation ability may influence the choice of emotional labor strategies through resource preservation logic.”

Red Tape in Business Contexts

At the end of the conceptual discussion of red tape, we added the following paragraph:

“While the concept of red tape originates from public administration, recent studies have demonstrated its applicability in business contexts, particularly in large chain retailers where internal procedures, documentation, and surveillance can resemble bureaucratic systems. In contrast, smaller retailers may encounter less formal red tape but still face operational inefficiencies from ambiguous practices or customer-related burdens. These differences highlight the need to further investigate red tape across different types of commercial organizations in future studies.”

**2.Refine Hypothesis Logic. The logic chain between H1 and H3—particularly the argument that resource depletion leads to less surface acting—contrasts with traditional models where emotional dissonance leads to more surface acting. It is recommended to strengthen the theoretical rationale for this divergent path and to streamline the language to better highlight the novelty of the authors’ perspective. Additionally, the interpretation that highly customer-oriented employees express authentic negative emotions seems speculative; more literature support or alternative emotion regulation strategies (e.g., cognitive reappraisal) may enhance this explanation.**

Response:

We appreciate this thoughtful critique and have strengthened the logic of our hypotheses as follows:

Clarifying the Resource Depletion → Reduced Surface Acting Pathway

We added the following explanation to explicitly distinguish our perspective from traditional dissonance models:

“This logic departs from traditional dissonance-based models, which assume employees always retain enough capacity for surface acting. In high red tape environments, employees may reach a ‘resource exhaustion threshold’ where even minimal effort to feign emotions becomes too costly. Under COR theory, individuals facing continuous resource loss tend to withdraw from optional demands, including superficial displays of compliance. Hence, the decline in surface acting can be interpreted as a strategic disengagement from emotionally taxing behaviors, rather than a failure to comply.”

Incorporating Cognitive Reappraisal for High Customer Orientation Employees

Just before Hypothesis 6, we introduced an alternative emotion regulation explanation:

“However, employees with high customer orientation may not always resort to surface acting when red tape disrupts their service delivery. Instead, they may engage in adaptive emotion regulation strategies such as cognitive reappraisal—reframing frustrating procedures as manageable challenges or temporary obstacles. This reframing helps conserve emotional resources and sustain deep acting or authentic emotional expression, rather than relying on inauthentic surface displays.”

**3.Increase Transparency in Methods。Some conditional indirect effects approach non-significance (e.g., in Table 4, the 95% CI for surface acting at high customer orientation includes zero). The authors should advise readers to interpret these results with caution and discuss potential biases due to sample or contextual limitations. Moreover, providing more detail on the localization of survey instruments (e.g., translation process, pilot testing) would strengthen the credibility and cultural relevance of the measurements.**

Response:

Thank you for highlighting these important concerns. We have addressed them as follows:

Caution Regarding Confidence Intervals

Following the discussion of Table 4, we added:

“It should be noted that the confidence interval for the conditional indirect effect of red tape on surface acting via negative emotions at high customer orientation includes zero. This suggests that the moderated mediation effect is not statistically robust in this condition, and readers should interpret this result with caution. The lack of significance may be attributed to sample size limitations or contextual variability.”

Survey Localization Process

After the description of the Red Tape scale, we included:

“The survey instruments were first translated from English to Chinese using a forward-backward translation method. Two bilingual experts conducted the initial translation and a separate group of experts reviewed the back-translation for semantic consistency. A pilot study with 35 frontline retail employees was conducted to assess item clarity and contextual relevance, leading to minor adjustments in wording.”

Sample and Generalizability Concerns

In the “Limitations and Future Directions” section, we added:

“The contextual specificity of post-pandemic Beijing retailing may also limit the generalizability of the moderated mediation results. Future studies should expand the sample to cover diverse cities and sectors, and incorporate multi-source data (e.g., managerial ratings or customer satisfaction records) to further validate the observed relationships.”
